# Using evidence-based guidelines to inform service provision: a structured mapping exercise within the National Health Service Diabetes Prevention Programme in England

**DOI:** 10.1186/s13104-018-3546-8

**Published:** 2018-07-27

**Authors:** Anna Haste, Linda Penn, Angela M. Rodrigues, Marta M. Marques, Kirsten Budig, Ruth Bell, Carolyn Summerbell, Martin White, Ashley J. Adamson, Falko F. Sniehotta

**Affiliations:** 1 Institute of Health & Society, Newcastle University, Baddiley Clark Building, Newcastle upon Tyne, NE2 4AX UK; 2 Fuse: UKCRC Centre for Translational Research in Public Health, Newcastle upon Tyne, UK; 3 Human Nutrition Research Centre, Newcastle University, Newcastle upon Tyne, UK; 4 MRC Epidemiology Unit, University of Cambridge, Cambridge Biomedical Campus, Cambridge, CB2 0QQ UK; 5 School of Applied Social Sciences, Durham University, 32 Old Elvet, Durham, DH1 3HN UK; 6 Department of Clinical, Educational and Health Psychology, University College London, 1-19 Torrington Place, London, WC1E 7HB UK

**Keywords:** Evidence-based guidelines, Structured mapping, Practical implementation, Diabetes prevention

## Abstract

**Objective:**

The National Health Service (NHS) in England planned a national diabetes prevention programme (NHS DPP) with phased implementation. Evidence-based guidelines and service specifications support efficient and effective translation of research into practice. We aimed to evaluate the use of a structured mapping exercise to appraise how evidence, service specification and early phase practice could inform recommendations to guide subsequent implementation of the NHS DPP.

**Results:**

The mapping exercise facilitated comparison and appraisal of key components from different documentary sources (evidence-based NICE guidelines, service specification, and provider documents). Key components were categorised into (A) pathways into programmes, (B) intervention content (C) inequalities and (D) quality assurance and staff training. We identified where key components were the same (accordance), where they varied (discrepancies) and where they were lacking (discontinuities), across the documentary sources. For example there was discrepancy in intervention duration and discontinuity in intervention enrolment procedures. This mapping exercise was useful to compare the fidelity in translation of evidence-based guidance into service specification and programme documents, thus identifying where future service implementation might be improved. This method may be applicable for use with other health conditions where research evidence requires translation into real world population programmes.

**Electronic supplementary material:**

The online version of this article (10.1186/s13104-018-3546-8) contains supplementary material, which is available to authorized users.

## Introduction

The NHS 5 year forward view in England emphasised the need for ‘a radical upgrade in prevention and public health’ and included a plan for a national diabetes prevention programme (NHS DPP) [1].

The NHS DPP in England, for individuals at high risk of developing type 2 diabetes (T2D), was planned to be rolled out in phases (i) demonstrator site phase (seven sites in England), (ii) wave 1 (four procured providers in 27 sites across England, permitting 20,000 referrals in 2016/17) and (iii) wave 2 (nationally to the whole country by 2020 with an expected 100,000 referrals available each year). The stated objectives were reduction in incidence of T2D, blood glucose parameters and weight [2].

The NHS DPP service specification [2] was developed by NHS England using research evidence reviews and reports [3, 4], input from an Expert Reference group, a User Involvement group and analysis of the Health Survey for England data. The demonstrator site phase relied mostly on applications from local health economies, where relevant services were already being delivered, and was intended to inform subsequent implementation of the NHS DPP.

National Institute for Health and Care Excellence (NICE) guidelines are created to improve outcomes for those using health services [5]. However, evidence-based guidelines do not necessarily result in the anticipated change in practice. Where guidance is available there are often gaps between evidence-based principles, contractual agreements around intervention commissioning and actual provision of services and interventions [6].

Translation of research into practice involves making sure research findings about effective treatments reach populations that can benefit and are implemented as intended [7]. Reflection on the guidelines available and how these are implemented in practice is necessary to make best use of the recommendations in an applied setting [8].

Summary of the process evaluation of the demonstrator and wave 1 phases of the NHS DPP are reported elsewhere [9].

We aimed to appraise how evidence informed practice to guide subsequent implementation of the NHS DPP through a structured mapping exercise [10].

## Main text

### Methods

To conduct the mapping exercise we reviewed and extracted data from all the relevant evidence/documentary sources. The documentary sources used within the mapping method were:

1. NICE guidelines—PH38 preventing T2D guidance for individuals at high risk [11].
2. The draft NHS DPP service specification (demonstrator site phase).
3. The final NHS DPP service specification [2] (wave 1 phase).
4. All of the seven demonstrator site applications and Memoranda of Understanding (MoU) submitted to become part of the NHS DPP demonstrator site phase. Any provided baseline documentation from the seven sites.
5. All of the four procurements and Memoranda of Understanding (MoU) submitted to become a provider for the NHS DPP wave 1 phase. Any provided baseline documentation from the four providers was reviewed.

Data was extracted from the above documentary sources in relation to Key components. Components related to the whole of the programme were extracted to enable the complete T2D prevention pathway to be reviewed and synthesised. These included:

A. Pathways into the programmes (identification, recruitment, referral, enrolment)
B. Intervention content (intervention components using existing reporting frameworks and taxonomies [12–14])
C. Inequalities using PROGRESS equality indicators (place of residence, race/ethnicity/language, occupation, gender/sex, religion, education, socioeconomic status, social capital) [15]
D. Quality assurance and staff training (fidelity measures, resources, staffing, training requirements)

Information was extracted on staff or health care professional involvement at each stage of the programme and also areas of responsibility, i.e. training of delivery staff.

Structured mapping was used to collate the evidence and enable comparison of the findings across the different documentary sources. Initially we used a spreadsheet to facilitate the mapping process and we used recommendations in NICE guidance (PH38) to identify key components [16]. The extracted data were then organised into tables (Table 1).

**Table 1:** Mapping of intervention content

Components	Documentary sources				
	NICE guidelines PH38	Demonstrator site phase		Wave 1 phase	
		NHS draft DPP specification	Collated demonstrator sites	NHS DPP specification	Collated wave 1 providers
Aims	Lifestyle-change programmes to provide advice and support on physical activity, weight management and diet	Behavioural intervention with three main goals (1) Dietary improvements (2) Physical activity (3) Weight reduction	Most programmes developed by demonstrator sites included the content components specified in NICE PH38 guidance and the NHS DPP service specification. Increasing physical activity was reported in six out of the seven sites; promoting weight loss in five out of the seven sites and improving dietary habits in six out of the seven sites	Behavioural intervention targeting: (1) weight loss (or maintenance), (2) achievement of UK dietary recommendations related to fibre, F&A, oily fish, saturated salt, and free sugars; (3) achievement of the CMO physical activity recommendations	All four providers included core sessions covering topics on type 2 diabetes and risk factors, weight loss and maintenance, diet, and physical activity. In agreement with recommendations, all sites provided a curriculum for their programmes with sufficient detail on the content of the sessions. All sites reported following UK weight loss, dietary and physical activity recommendations outlined within the NHS DPP specification
Format	Groups of 10–15 people or one-to-one basis or mixture Run at different times and days (evenings/weekends)	Group sessions, face-to-face though individual can also be included	The majority of demonstrator sites programmes were delivering interventions in a mixture of group sessions, face-to-face sessions, and individual sessions. One site was an exception using a telephone-based intervention. Two sites also included a digital component in their programmes	Group sessions, face-to-face. Max. 20 part. Individual sessions can also be included. Sessions to be delivered in a format, at times and venues that are appropriate for different groups in the community (e.g. weekend). Family of peer support accommodated where helpful for user	All providers had group-based in-person. Two reported the acceptance of accompanying friends or family members
Additional contact	Not mentioned	Not mentioned	Two sites included a digital component in their programmes	For non-face-to-face contact, details on this should also be provided	One provider used additional telephone support (number of sessions or frequency not specified). Another provider used remote support (text messages, email, telephone, and social media) and another incorporated individual in-person sessions
Duration	At least over a period of 9–18 months; Follow up sessions every 3 months for at least 2 years	Across 9 months minimum	Varied greatly with some being only 6 weeks and others lasting up to 12 months. Only three demonstrator sites were compliant with the recommended duration	Standardised across providers Minimum of 9 months	Duration varied between the providers, ranging from 6 months up to 12 months (with 6 months follow up). Only one provider did not comply with the 9 months minimum duration recommendation
Intensity	At least 8 sessions (minimum of 16 h); weekly or fortnight sessions; reduce intensity over time	Series of sessions At least 13 sessions, with minimum contact of 16; 1–2 h sessions	The intensity of interventions differed greatly between sites. The intensity of sessions across the DPP programmes varied from six sessions to 52 sessions, with some sessions being held twice a week and others monthly	Standardized across providers At least 13 sessions with a minimum total of 16 h contact time; 1–2 h session Brief interventions can be classified as session delivered if above 13 sessions of 1–1 h (or for e.g. 13 1–2 h with 4 brief interventions in addition, totalling 17 sessions) Final session counts as toward intervention hours (not the assessment session)	The intensity of interventions differed between sites. The number of sessions varied between 11 and 18, with one provider not meeting the recommended number of sessions. Length of individual sessions ranged from 60 to 90 min in line with the recommendations
BCTs	Information provision Motivational interviewing Goal setting Action planning Coping plans Relapse prevention Self-regulation techniques (e.g. self-monitoring)	Include, but not limited to, goal setting and self-monitoring	There was limited information about use of behaviour change techniques (BCTs) in baseline documents from demonstrator sites. Two sites mentioned using information provision, motivational interviewing, action planning, coping planning, relapse prevention, self-regulation techniques; goal setting; four sites described the use of goal setting and both motivational interviewing and action planning in their programme; and social support was reported in one site	Use of BCTs standardized across providers Provider must be explicit re the techniques (BCTs) used, as well as the expected mechanism of action Framework must be used to detail this info Include, but not limited to, goal setting and self-monitoring For non-face-to-face contact, details on this should also be provided	All sites used the BCT Taxonomy V1 [14] and theory-driven techniques and linked these techniques with the expected mechanisms of action. In addition, all sites used the recommended BCTs, and most used additional evidence-based techniques for sustained behaviour change

*BCTs* behaviour change techniques

The mapping exercise drew on Structured Mapping Theory, which describes the use of mapping and how evaluation of the analogy gives a measure of the quality of match between the base and a target [10]. Critical appraisal identified whether key components across and between the documentary sources were in:

1. Accordance—components that were common and reported across all documentary sources, e.g. the format of the intervention (face-to-face group sessions).
2. Discrepancies—components that varied across documentary sources, e.g. duration or intensity of the intervention.
3. Discontinuities—components that did not appear across all documentary sources, e.g. intervention enrolment procedures.

We used the Accordance, Discrepancies, Discontinuities (ADD) ‘ADD-Fuse’ method outlined above, which was developed during the NHS DPP demonstrator and wave 1 phase evaluation projects, to facilitate critical appraisal. Critical appraisal identified where programmes or specifications consistently met the desired criteria or where differences or gaps were present and therefore where improvements could be recommended. Recommendations were formulated from the appraisal process and provided to the NHS DPP management team to inform subsequent phases (Additional file 1). Using this mapping exercise on two different phases of the NHS DPP showed how the programmes and service specifications progressed between these phases.

**Fig. 1:**
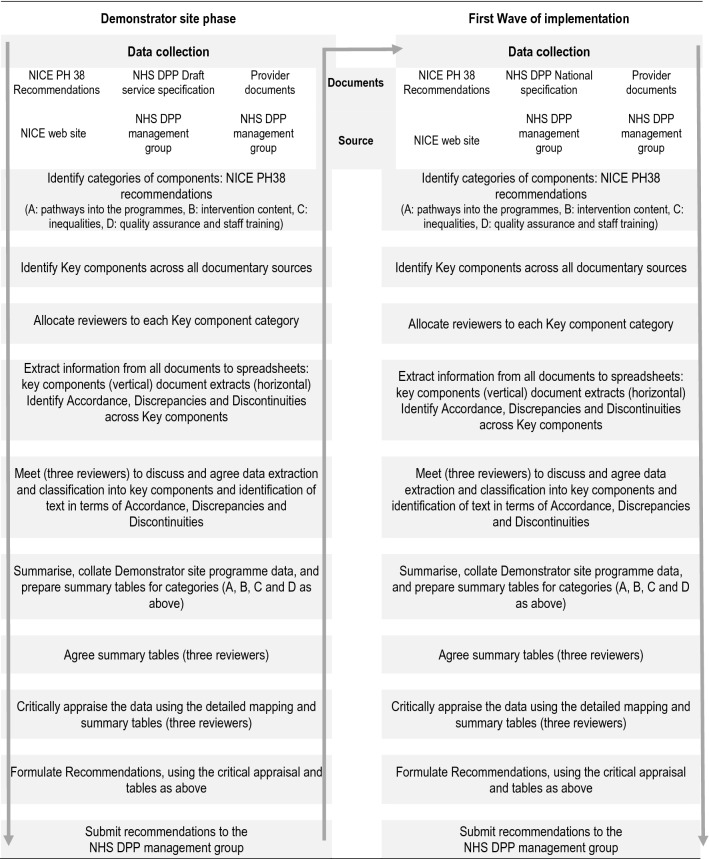
Flow chart of data collection and methodology: ADD-Fuse method applied to the NHS Diabetes Prevention Programme in England

The mapping exercise was completed independently by two reviewers with expertise in behaviour change interventions and checked by a third reviewer in both phases, any disparities were resolved through discussion. However, we found that the clear specification of key components and the agreed classification as Accordance, Discrepancy and Discontinuity for each key component across each documentary source led to a high degree of consistency between reviewers. The data collection and methodology are summarised in a flow chart (Fig. 1).

### Results

Table 1 provides an example of how the mapping exercise was conducted.

Table 1 illustrates how the mapping exercise facilitated the identification of key components, actors and responsibilities within the NICE guidelines, NHS DPP service specification and NHS DPP provider documentation (the applied setting/context). Tables were then used to compare and contrast across the different documentary sources.

We described this method as identifying Accordance, Discrepancies and Discontinuities (the ADD-Fuse method), which was used to highlight the key commonalities, differences and gaps between the documentary sources (Table 2).

**Table 2:** Using Accordance, Discrepancies and Discontinuities (ADD-Fuse) to identify service specification recommendation

	Demonstrator site phase of NHS DPP		Wave 1 phase of NHS DPP	
	Component of the programme	Recommendation	Component of the programme	Recommendation
Accordance	Format: Face-to-face group sessions were suggested for the format	Format was being implemented as recommended	Format, content and aims: Face-to-face group sessions were suggested for the format, with the core sessions/aims recommended to focus on weight loss, diet and PA	The format and the three core goals of the programme were followed as recommended
Discrepancy	Duration and intensity: Minimum of 9 months. At least 13 sessions, with minimum contact of 16; 1–2 h sessions	Intensity and duration varied greatly between demonstrator sites with some not achieving the recommended intensity or duration. Systems for collecting data to assess intensity and duration, such as attendance and contact details, should be considered	Duration and intensity: Standardised across providers Minimum of 9 months. At least 13 sessions, with minimum contact of 16; 1–2 h sessions	Duration and intensity still remained varied, however only one provider reported less than the minimum specification recommendations. Further monitoring and development of programmes with providers is needed to improve specification achievement, which at present could impact on programme outcomes
Discontinuity	BCT description: Include, but not limited to, goal setting and self-monitoring	There was limited information about the use of behaviour change techniques (BCTs) in baseline documents from demonstrator sites. More specific information about the use of BCTs should be included in the NHS DPP specification, especially as there is currently a discrepancy between NICE PH38 guidance that recommends specific BCTs and the NHS DPP specification that provides limited guidance on the use of BCTs	Additional contacts: For non-face-to-face contact, details on this should also be provided	Detail of the additional contacts providers incorporated with patients was minimal. Greater detail on the aim and content of the non-face-to-face contact and materials used is needed. The service specification could develop a checklist to support providers

Table 2 illustrates how the identification of accordance, discrepancies and discontinuities (ADD-Fuse method) led to the formulation of recommendations for improvements in relation to the NHS DPP service specification, the planned implementation of the DPPs (provider documents) or both. Recommendations were provided to the NHS DPP Management team and responses to the recommendations were received from the Management team back to the research team (Additional file 1). This method identified key components in the service specification that impact on implementation.

#### Accordance

In both the demonstrator and wave 1 phases, the format of the intervention was in accordance with the NHS DPP service specification (in person group sessions).

#### Discrepancy

The reporting of the content of the face-to-face sessions, the level of detail on outcomes, mechanisms of action and techniques used varied greatly between providers. As outlined in Tables 1 and 2 discrepancies were identified in the duration and intensity of the intervention provided in both phases (demonstrator and wave 1). One wave 1 provider did not meet the required standard for duration and intensity, which varied across the four providers. This variation poses an issue for outcome evaluation across the provider interventions. Therefore monitoring of patient contacts is vital to ensure clarity in intervention provision and the impact of this on intervention outcomes.

#### Discontinuity

A gap (discontinuity) in the draft NHS DPP service specification was identified at the demonstrator phase for the description of behaviour change techniques (BCTs) when compared with recommendation s in NICE guidelines (Table 1). However, by wave 1 more detailed BCT description was requested in the NHS DPP service specification, all providers incorporated the recommended BCTs, and most used additional evidence-based techniques for sustained behaviour change. Detail on additional contact with patients (i.e. telephone support, text messages or social media contact) outside of the standard in group sessions was an identified discontinuity in wave 1 provider documents. The remote contact and materials used, including digital components, should be described with the same level of detail as the other components, including reference to the specific behavioural outcomes, theoretical basis and techniques used. While this level of detail was recommended in the NHS DPP national service specification, the providers did not provide it in such detail.

### Discussion

#### Key findings

Evidence-based documentary sources were used to examine incorporation of evidence in the planned context of the NHS DPP programme. Comparison identified accordance, discrepancies and discontinuities (ADD-Fuse method). Different components, actors and responsibilities that may impact the implementation and evaluability of the NHS DPP were revealed. This process identified recommendations (Additional file 1), informing subsequent phases of the NHS DPP, as to where further clarification and consideration was required to either improve the service specification and/or support the transition of evidence into practice.

#### Comparison with other studies

Evidence-based lifestyle interventions to prevent or treat diabetes have been shown to be effective and have the potential to reduce morbidity and mortality rates [17–20]. A difficulty in translating DPP’s into practice is the need to adapt to all patients, clinicians or setting needs. As all local services need to adapt for the diverse UK population it is vital to monitor intended variations as well as unintended variations that occur during implementation, highlighting the importance of process evaluations [21]. A previous review identified translational strategies and cultural adaptations were frequency used to in order for DPP’s to reach diverse populations and those from disadvantaged socioeconomic backgrounds, e.g. adapting materials (including information on local foods or traditional physical activities), reducing the frequency of classes or using community health workers to deliver classes. This review stated how adaptations often go unreported and supports the use of a structured approach to documenting translation, as offered in this current manuscript, to facilitate identification of implementation and effectiveness [22].

#### Strengths

Mapping two stages of the NHS DPP (demonstrator phase and wave 1) made it possible to trace the progression of a new service as the phases were rolled out in England and observe changes in the NHS DPP service specification over time. The mapping exercise evaluated the programme as a whole, informing on wider aspects of a health improvement programme that could be improved, which would not be assessed if focused solely on the intervention.

#### Implications

Variance in delivered programmes is likely to have an impact on the assessed outcomes. This structured mapping exercise has utility for implementation science and real-world programmes in explaining differences in outcomes based on specific components of the interventions and how each programme is implemented in relation to the service specification. This method could also enable the identification of key areas that require improvement. The mapping exercise examined the progression of a national programme rollout, identifying how the service specification developed from a draft to a final document (e.g. incorporating greater detail on the inclusion of BCTs and addressing inequalities). This mapping exercise could be utilised in further rollout of the NHS DPP. This process could be used for the development of future service specifications and in the reporting of behaviour change programmes. Fidelity measures need to be established in order to judge whether implementation of a programme meets the required standards.

### Conclusion

A mapping exercise was applied in the context of the NHS DPP in England. Using NICE guidelines allowed the service specification and provider documents to be examined in relation to the evidence base. This enabled identification of whether the implementation of a new health care programme may experience problems owing to shortcomings in the service specification or whether problems lie within the transition from evidence into practice. We suggest the method may be applicable for use within other disease or health conditions where research evidence requires translation into real world population programmes.

## Limitations

The strength of the evidence base varies across different health conditions and therefore using a mapping exercise like this may not be applicable to programmes that do not have existing evidence-based guidelines and where the evidence is minimal or of poor quality.

The NHS DPP explicitly entered other sources of evidence into the specification development (users, experts, new evidence syntheses) and this has implications for how closely the programme tracks the research evidence. There are of course reasons for doing this, for example practicality and funding can impact greatly, but this brings risks that the key components that make an intervention effective become diluted.

Since the mapping exercise additional evidence has become available, in particular the 2017 update to the NICE PH38 guidelines [23]. Data extraction relied on information provided from demonstrator site and wave 1 providers.

## Additional file

**Additional file 1.** Recommendations provided to the NHS DPP Management team and responses received from the NHS DPPManagement team.

## Abbreviations

BCTs: behaviour change techniques
DPP: diabetes prevention programme
NHS: National Health Service
NHS DPP: National Health Service Diabetes Prevention Programme
NICE: National Institute for Health and Care Excellence
T2D: type 2 diabetes
