# Supplementary material for: Using evidence-based guidelines to inform service provision: a structured mapping exercise within the National Health Service Diabetes Prevention Programme in England
Source: BMC Res Notes. 2018 Jul 27;11:510. doi: 10.1186/s13104-018-3546-8 (PMC6064162; doi:10.1186/s13104-018-3546-8)
Supplement: Supplementary file 1 — Additional file 1. Recommendations provided to the NHS DPP Management team and responses received from the NHS DPPManagement team. [file 13104_2018_3546_MOESM1_ESM.docx]

**Diabetes Programme Board 6^th^ March 2017**

**Item 7**

| **Author** | Jenifer Smith |
| --- | --- |
| **Date** | 02/03/17 |
| **Version** | v2 |

Response to FUSE Evaluation Findings

Background

1. The Healthier You: NHS Diabetes Prevention Programme is the first nationally delivered behavioural intervention to prevent the onset of Type 2 diabetes in the world. The urgent need to introduce such a programme was set out in the Five Year Forward View, and health surveillance statistics confirm that there is a continued need for such action. Policy was made, and the current programme designed, on the basis of the best available evidence of cost effective behavioural interventions to prevent or delay the onset of Type 2 diabetes. Such an approach has secured the traction across the clinical community which is needed for successful implementation. Sustaining this clinical engagement would be facilitated by further developing the programme in the light of evidence of effectiveness acquired though evaluation of the service operation during implementation, and of the longer term outcomes of participants. Future commissioning decisions will be made in the light of policy analysis, operational efficiency and effectiveness, and the short and long term health outcomes for participants.
2. Demonstrator sites were established to explore the effectiveness of service delivery models and functional elements within these. The Department of Health commissioned FUSE, The Centre for Translational Research in Public Health, to undertake an evaluation of the demonstrator sites for the NHS Diabetes Prevention Programme. This was to be completed by April 2017, with a view to informing future commissioning, and superseded by a formal evaluation of the entire NHS Diabetes Prevention Programme commissioned via the National Institute for Health Service Research. At a recent meeting with the researchers from FUSE it was agreed that the NHS Diabetes Prevention team would review and comment on the outputs from the evaluation. It would be reasonable for these to be considered in the light of the evolution of the diabetes prevention programme and future commissioning decisions.
3. This paper considers the outcome available to date from the work by FUSE, the scope of which was extended to include available information from the early implementers of the nationally commissioned intervention.
4. The Diabetes Programme Board is asked to note the proposed response to the recommendations made by FUSE.

Findings

1. The evaluation covers the demonstrator sites and, as would be expected, in many cases the contract specification for the NHS Diabetes Prevention Programme encompasses the findings and recommendations. In other cases the findings provide further confirmation of experience in the early phases of implementation, against which action has already been taken. In the light of this the table set out in Appendix A categorises the recommendations as follows:

A) recommendations implemented before the evaluation report was received;

B) recommendations implemented in response to the evaluation;

C) Recommendations that might be considered for the next round of procurement;

D) Recommendations deferred.

Discussion

1. The findings of the FUSE report should be considered within the context of the functions undertaken by the Programme Board which should be influenced by evidence from evaluation. These could be broadly defined as policy formulation, service specification, definition of service delivery model, and commissioning. To be of practical use, further evidence needs to be available within a timeframe which aligns with the political framework and the NHS business planning cycle, so that it is available at the point where key decisions on policy design and resourcing are made. Achieving such alignment with research commissioned through government funded sources is challenging, but to achieve optimum value from public resource, it behoves us to consider to what extent this research was appropriately aligned and whether we could make any suggestions for improvement and whether there are any points of learning from the process which may be relevant, particularly in the light of the National Institute for Health Research commission.
2. We would make the following observations about the FUSE work:

- The service specification had to be defined prior to procurement which was well in advance of the anticipated output from FUSE. Hence some evaluation of the processes under study in demonstrator sites had to take place internally; whilst external triangulation of this is welcome, it reduces the value of the findings somewhat and limits ability to respond to them in the current contracting cycle.
- Service delivery was (as is often the case) the area for which there is a limited evidence base and the extent to which service models from elsewhere can be applied in England can only be answered by real-time study. This is an area where the FUSE research could make a significant contribution, provided the research teams are well aligned with the service. On reflection this could have been improved, and should be addressed at the outset with any future commissioned work. The research team need access to data being generated through the programme and to personnel delivering the service, whilst the Diabetes Programme Board needs early access to findings so that these inform key decision points in the business planning cycle to facilitate maximum relevance of the research output and early implementation for the benefit of patients.

Conclusion

1. The FUSE evaluation triangulates information from other (internal) sources and provides reassurance that the significant findings from the demonstrator sites have been incorporated in the specification for the NHS Diabetes Prevention Programme. It also identifies issues around information governance and the need to identify routes of recruitment to inform future commissioning decisions which are also emerging from our engagement with stakeholders as implementation continues, and some areas it would be helpful to get further information on through formal evaluation of the programme or some bespoke with service users. The recommendations on data items to be collected are, in part, more relevant to the evaluation of outcome from the programme than operational need for delivery. A balance will need to be agreed with the relevant stakeholders as to whether the cost of collection is justified in terms of the intended use and benefit to the programme as a whole.
2. We would welcome an early discussion with the successful research team for the NIHR evaluation to establish clear governance arrangements, lines of communication and an agreed publication schedule between ourselves.

**Annex A: Proposed Response to FUSE Recommendations**

|  | **Finding** | **Owner** | **Category (see key)** | **Proposed response** | **Action** |
| --- | --- | --- | --- | --- | --- |
|  | **Section A. Awareness raising and recruitment pathways, case finding, referral and enrolment** | | | | |
| **1** | In developing awareness raising and marketing strategies for the NHS DPP, consideration should be given to the different audiences and how strategies might be tailored for these audiences | PDG;  Framework Providers | A | This is a requirement of the NHS DPP service specification. | Further consideration to be given to segmentation in areas of poor uptake. |
| **2** | Responsibilities, for enrolment in intensive lifestyle change intervention should be made clear in the NHS DPP national specification. We suggest that responsibility for intensive lifestyle change intervention (ILCI) enrolment, including liaison with health care, should rest with ILCI providers. Appropriate staff training and support to fulfil this role should be made available. | PDG; local health economies | A | This is a requirement of the NHS DPP service specification. The specification makes it clear that providers are responsible for providing training for their staff. |  |
| **3** | Detailed description, analysis and evaluation of a variety of different recruitment routes and strategies would improve the NHS DPP national specification and inform its effective implementation. |  | A/C | 4 Pilots for direct recruitment undertaken; learning disability and gestational diabetes planned. |  |
| **4** | Use of HbA1c should be considered as the standard blood test eligibility criterion by the NHS DPP Management Group | PDG; Expert Reference Group | A/C | This is the intention but account has to be taken of local arrangements. | Continue to encourage referral on the basis of HbA1c |
| **5** | Specification of a standard risk-score for use in risk assessment would improve consistency in assessment of eligibility for the NHS DPP | PDG; Expert Reference Group | A | We do not agree as it is the responsibility of local delivery partners to identify ‘at risk’ people. Further, there is no “gold standard” risk assessment tool. We have worked to get validated tools used in NHS Health Checks. |  |
| **6** | Reliance on an over-riding single risk parameter (e.g. a blood test parameter alone) could be problematic in the NHS DPP specification. | PDG | A | We do not agree as this is a service available to those with non-diabetic hyperglycaemia and supply exceeds need |  |
| **7** | Consideration should be given to optimal methods and requirements for risk communication in the NHS DPP specification. | PDG; Providers | A | Providers are required to tailor communication of risk to users. | Ask this to be considered in formal evaluation specification and pursue with service users. |
|  | Section B. Intensive lifestyle change intervention: components, design and delivery | | | | |
| **8** | More detailed content specification is needed to improve consistency and fidelity of delivery of the NHS DPP intervention and facilitate evaluation. | PDG | D | There is a balance between core specification and tailoring to local demography. We want to consider this recommendation further as data on outcomes begins to emerge from the nationally procured service. | Monitor KPIs by provider and outcome from formal evaluation |
| **9** | Clear and detailed criteria regarding requirements for intensity and duration should be included in the NHS DPP specification. | PDG | A | This is specified in the contract. Further detail might be included in future procurements if an evidence base to justify this begins to emerge. |  |
| **10** | We suggest the use of phone or digital ILCI should be considered either as a standalone option in selected areas, or to augment face-to-face intervention provision. | PDG | A | A digital workstream is in progress |  |
| **11** | Specific ILCI components and general targets for behaviour change or health related outcomes should be clearly stated in the specification and acknowledged by intervention providers. | PDG | A | This is a contractual requirement |  |
| **12** | Greater clarity with regard to tailoring for weight management, physical activity and diet should be included in the national specification. | PDG | A | This is a contractual requirement |  |
| **13** | More specific information about the use of Behaviour Change Techniques (BCTs) in ILCI should be included in the NHS DPP specification | PDG | D | There is a requirement for Providers to specify their approach to behaviour change. Further detail might be included in future procurements if an evidence base to justify this begins to emerge. | Monitor KPIs by Provider and output from programme evaluation. Consider further specification to optimise outcomes. |
|  | **Section C. Inequalities and adaptation** | | | | |
| **14** | Men only and women only ILCI groups, with same sex intervention delivery staff and secure environment, should be required as available options for intervention delivery in the NHS DPP national specification. | PDG; local health economies | D | Local tailoring is required; gender specific groups may be appropriate | Monitor KPIs and output from programme evaluation and consider further specification if appropriate to optimise outcomes |
| **15** | Greater commitment to communicate appropriately with ILCI participants across diverse UK BME population groups is important to include within the NHS DPP specification. | PDG | A | Tailored communication is a contractual requirement |  |
| **16** | Inequalities should be specifically addressed in the NHS DPP national specification | PDG | A | KPIs address this |  |
|  | **Section D. Quality assurance, data collection, monitoring and staff training** | | | | |
| **17** | It is important that the data collected make it possible to identify whether the NHS DPP has an effect on key health related outcomes such as HbA1c and weight. | PDG | A | Contract MDS in place. |  |
| **18** | It is also important to collect and analyse data on uptake and retention in relation to different recruitment routes and population sub-groups. | PDG | A | Contract MDS in place |  |
| **19** | When sending invitations to patients it appeared to be more effective if people received the invitation from their general practitioner as a figure of authority that patients would heed. More involvement of GPs in the referral process was suggested by deliverers, but future evaluations should also monitor the impact of the NHS DPP on GPs workload | PDG | C | GP involvement is recognised as beneficial. To date the majority of referrals into the programme have been initiated by GPs. | Consider including impact on GP workload in specification for programme evaluation. |
| **20** | When referring a patient to the NHS DPP the importance of enrolling people quickly, once a referral had taken place and ensuring the programme took place in a nearby locality with flexible timings was highlighted. Referral into alternative services if the NHS DPP was not suitable or if patients were not eligible was also suggested. | PDG | A | This is recognised. There are KPIs in place in relation to speed of referral and a requirement that eligible individuals who do not accept an invitation to enrol on the Service are given information about the NHS Choices website pages related to weight management, physical activity and healthy lifestyles and to any other locally available resources for supporting weight loss, healthy eating and physical activity. | Continue to monitor KPIs and consider if further specification of this is required in further procurements. |
| **21** | The need to make effective use of the first opportunity to inform people at risk that changes can be made to avoid type 2 diabetes, and that it is not necessarily inevitable, emerged as an important theme. Providing a full explanation of the condition and their individual diagnosis was viewed as essential so that people understood why they have been referred and the benefit of attending the programme | PDG and Providers | A | Part of specification. |  |
| **22** | The need for more guidance about sessions’ content and the NHS DPP curriculum, whilst ensuring enough flexibility to address any cultural sensitivities or tailor to users’ preferences/needs was an important finding from interviews with deliverers | PDG | C |  | This may be important as the digital workstream progresses. |
| **23** | Service users emphasised the need for blood test results to be recent and shared effectively amongst the relevant health care professionals and intervention delivery staff. The use of outdated blood test results could lead to service user confusion and incorrect information being provided to deliverers | PDG; Providers | A/B/C | There is a requirement for recent blood test on referral. We are working to reduce confusion on eligibility and conflicting blood results. | Continue to seek service user feedback. |
| **24** | Clarity is needed on responsibilities within each part of the NHS DPP. Commissioners suggested that responsibilities should be written into provider contracts (i.e. who should be responsible for referring eligible patients to the NHS DPP, who should be responsible for organising training for deliverers, who should be responsible for collecting end of intervention patient data). Active involvement in the NHS DPP implementation process with more opportunities to feed back to the management was also highlighted by stakeholders (i.e. commissioners and intervention deliverers | PDG and local health economies | A/B | Responsibilities are specified in the contract and kept under review. | Continue to seek feedback from providers and users to identify and resolve any outstanding confusion on responsibilities. |
| **25** | Improvement in data sharing agreements and permissions were considered desirable in order to ensure participants’ progress could be tracked throughout the pathway and their outcome/progress is fed back to any relevant stakeholders. | PDG | A/B | Data sharing is always subject to negotiation and compliance with information governance protocols. |  |
